# Improving quality in adult long covid services: Findings from the LOCOMOTION quality improvement collaborative

**DOI:** 10.1016/j.clinme.2024.100237

**Published:** 2024-08-23

**Authors:** Julie Darbyshire, Trisha Greenhalgh, Nawar D. Bakerly, Kumaran Balasundaram, Sareeta Baley, Megan Ball, Emily Bullock, Rowena Cooper, Helen Davies, Johannes H. De Kock, Carlos Echevarria, Sarah Elkin, Rachael Evans, Zacc Falope, Cliodhna Flynn, Emily Fraser, Stephen Halpin, Samantha Jones, Rachel Lardner, Cassie Lee, Ashliegh Lovett, Victoria Masey, Harsha Master, Ghazala Mir, Adam Mosley, Jordan Mullard, Rory J. O'Connor, Amy Parkin, Anton Pick, Janet Scott, Nikki Smith, Emma Tucker, Paul Williams, Darren Winch, Conor Wood, Manoj Sivan, LOCOMOTION consortium

**Affiliations:** a Nuffield Department of Primary Care Health Sciences, University of Oxford, Oxford, UK; b Northern Care Alliance NHS Foundation Trust, UK; c University Hospitals of Leicester NHS Trust, UK; d Person with lived experience of long COVID and Birmingham Community Health Care NHS Foundation Trust, UK; e Newcastle Upon Tyne Hospitals NHS Foundation Trust, UK; f Research, Development & Innovation Division, NHS Highland, Inverness, UK; g Cardiff & Vale University Health Board, UK; h Research, Development & Innovation Division, NHS Highland UK, North West University, Potchefstroom, South Africa; i Imperial College Healthcare NHS Trust, UK; j University of Leicester, UK; k Birmingham Community Health Care NHS Foundation Trust, UK; l Oxford University Hospitals NHS Foundation Trust, UK; m Leeds Community Healthcare NHS Trust, UK; n Oxford Health NHS Foundation Trust, UK; o Hertfordshire Community NHS Trust, UK; p University of Leeds, UK; q University of Newcastle, UK; r Research, Development & Innovation Division, NHS Highland Inverness, UK; and MRC-University of Glasgow Center for Virus Research, University of Glasgow, Glasgow, UK; s Person with lived experience of long COVID and member of the LOCOMOTION Patient Advisory Group

**Keywords:** Covid-19, Long covid, Post covid-19 syndrome, Post covid-19 condition, Quality improvement collaborative, Postural orthostatic tachycardia syndrome, Breathing pattern disorder, Post-exertional malaise, Post-exertional symptom exacerbation

## Abstract

The protracted form of COVID-19 known as ‘long covid’ was first described in 2020. Its symptoms, course and prognosis vary widely; some patients have a multi-system, disabling and prolonged illness. In 2021, ring-fenced funding was provided to establish 90 long covid clinics in England; some clinics were also established in Scotland and Wales. The NIHR-funded LOCOMOTION project implemented a UK-wide quality improvement collaborative involving ten of these clinics, which ran from 2021 to 2023. At regular online meetings held approximately 8-weekly, participants prioritised topics, discussed research evidence and guidelines, and presented exemplar case histories and clinic audits. A patient advisory group also held a priority-setting exercise, participated in quality meetings and undertook a service evaluation audit. The goal of successive quality improvement cycles aimed at changing practice to align with evidence was sometimes hard to achieve because definitive evidence did not yet exist in this new condition; many patients had comorbidities; and clinics were practically constrained in various ways. Nevertheless, much progress was made and a series of ‘best practice’ guides was produced, covering general assessment and management; breathing difficulties; orthostatic tachycardia and other autonomic symptoms; fatigue and cognitive impairment; and vocational rehabilitation. This paper summarises key findings with the frontline clinician in mind.

## Introduction

Long covid is a widely-used term, usually referring to symptoms which persist more than 3 months after acute infection with SARS-CoV-2^1^; it is also known as ‘post-covid condition’^2^ or ‘post-covid-19 syndrome’.^3^ It occurs in 8–12% of people who were fully vaccinated at the time of their initial COVID-19 illness;^4^, ^5^, ^6^ it is more common, and tends to be more severe, in those who were unvaccinated at the time of that episode, especially if they were hospitalised.^5^^,^^7^^,^^8^

Long covid is a multi-system disease whose manifestations are multiple and varied.^9^ The Office of National Statistics Survey in March 2023 (the last date on which such statistics were collected) estimated that 1.8 million UK adults still had symptoms 12 weeks or more after acute COVID-19 infection; 20% described their symptoms as ‘severe’ .^5^ In approximate order of frequency, symptoms include fatigue, shortness of breath, cognitive impairment (‘brain fog’), muscle and joint pain, chest pain, palpitations, persistent loss of smell and taste, gastrointestinal upset, headache, rashes and other allergic symptoms, anxiety and depression.^7^, ^8^, ^9^, ^10^ Pre-existing comorbidities may include asthma, allergies, musculoskeletal conditions, insomnia, headaches, chronic fatigue, mental health conditions and attention deficit-hyperactivity disorder, and may be exacerbated in long covid.^7^, ^8^, ^9^, ^10^ Deconditioning and sarcopenia often accompany (but are rarely the underlying cause of) long covid.^11^

The natural history of long covid in most patients is gradual recovery,^7^^,^^10^, ^11^, ^12^ but in some it relapses and remits, with characteristic ‘crashes’ (exacerbations of symptoms including fatigue and cognitive impairment, also known as post-exertional symptom exacerbation or PESE) following physical, mental or emotional stress.^13^ The risk of thrombotic complications (cardiac, respiratory and neurological) is raised for months and possibly years after acute COVID-19 infection.^7^^,^^14^ The chance of full recovery diminishes the longer the patient has had long covid, partly because such patients seem vulnerable to reinfection.^15^

Research into the causes of long covid has progressed rapidly and produced numerous hypotheses involving viral persistence or reactivation, immune dysregulation, autonomic dysfunction, endothelial inflammation and immune-thrombosis, and altered gut microbiome,^16^, ^17^, ^18^, ^19^, ^20^ but advances in the basic science of long covid have not yet translated into clinical therapies. At the time of writing, the cornerstone of management is holistic assessment and investigation to assess severity, assess and manage comorbidities and exclude thrombotic complications, followed by whole-patient rehabilitation by a multidisciplinary team (MDT). The latter would ideally include pacing strategies (avoiding post-exertional crashes), physiotherapy (especially breathing exercises), occupational therapy (for cognitive and vocational rehabilitation), psychological support, plus speech rehabilitation, olfactory training and dietary advice as needed.^21^

In October 2020, policy recommendations for long covid services in England proposed the introduction of long covid clinics via a tiered service. ‘Tier 1′ referred to supported self-management; ‘tier 2′ was generalist assessment and management in primary care; ‘tier 3′ was specialist rehabilitation or respiratory follow-up with oversight from a consultant physician, and ‘tier 4′ was tertiary care for patients with complications or complex needs.^22^ In 2021, ring-fenced funding was allocated to establish 90 multidisciplinary long covid clinics in England.^23^ Some clinics were also set up in Scotland and Wales with local funding. Clinics varied widely in eligibility criteria, referral pathways, staffing mix (some had no doctors at all), and investigations and treatments offered.

A further policy document on improving long covid services was published in 2022^24^; it recommended that specialist long covid clinics with MDT care should continue; that clear referral pathways were needed from both primary care and inpatient care (e.g. following admission for acute covid-19); that patients should be monitored using PROMs (patient-reported outcome measures); and that unwarranted variation (e.g. in waiting times by geographical region) and inequalities in access and outcomes should be addressed. New commissioning guidance was published in December 2023.^25^

The empirical study reported here began soon after the first of the above policy documents was published and ended just as the last was published. The aims were:

1. To optimise access, assessment, monitoring and management in long covid specialist clinics using a quality improvement collaborative model; and
2. To produce guidance that would be useful to non-specialists, especially those managing long covid in primary care.

## Methods

The study was one work package of the wider LOCOMOTION (Long Covid Multidisciplinary consortium Optimising Treatments and services across the NHS) research project involving 10 long covid services (eight in England, one in Wales and one in Scotland), which sought to optimise frontline long covid care in the UK National Health Service (NHS). The protocol for LOCOMOTION has been published.^26^ The 10 sites are summarised in Table 1; further details are given in the Supplementary Materials. The study is sponsored by the University of Leeds and approved by Yorkshire & The Humber— Bradford Leeds Research Ethics Committee (ref: 21/YH/0276). Trial registration number NCT05057260, ISRCTN15022307. Patient and staff participants gave written informed consent.

**Table 1:** Details of participating sites.

Site (jurisdiction)	Brief description
Site A (England)	GP-led clinic based in a large conurbation, linked to a teaching hospital. Large core MDT of allied professionals. Hybrid model (virtual or face-to-face). Close links to chronic fatigue service. Can refer on to hospital rehabilitation service.
Site B (Wales)	Hospital-based clinic run by a respiratory physician and clinical research fellow. No formal MDT support but close links with community recovery team.
Site C (England)	Entirely virtual community-based clinic jointly led by GP and OT and with large MDT of allied professionals. Complex patients are reviewed by the GP and referred on to secondary care specialties as needed.
Site D (Scotland)	Hospital and virtual clinic led by a clinical psychologist with a small MDT (infectious diseases consultant, OT, physiotherapist) The infectious diseases consultant also deals with post-treatment Lyme Disease in a separate clinic. Mostly virtual (video/phone) but can bring patients in for face-to-face or inpatient assessment if needed.
Site E (England)	Hospital clinic led by respiratory consultant with small MDT (psychologist, OT, physiotherapist). Primarily assessment service with referral for specialist input. Links with community-based rehabilitation service.
Site F (England)	Based in a community health service on the outskirts of a university city. Co-led by allied health professionals with large MDT including physiotherapy, OT, SLT, nursing, dietetics, psychology, GP and links to rehabilitation, respiratory and cardiology consultants and community mental health. Equally split between virtual, face-to-face in-clinic assessment and online group sessions, plus some home visits. Strong emphasis on rehabilitation and research.
Site G (England)	Hospital service based in the respiratory outpatient department in a tertiary hospital located on the outskirts of a university city. Originally a post-hospital follow-up clinic. Delivered by a large MDT with a weekly cross-specialty virtual meeting.
Site H (England)	Originally established as a respiratory follow-up clinic based in a large teaching hospital. Has evolved to become a comprehensive assessment clinic, amalgamated with CFS/ME service with referral pathways to other secondary care services. A nearby tertiary clinic is run by a cardiologist with a special interest in dysautonomia.
Site I (England)	Hospital service based in a rehabilitation department, co-located with various university research institutes, led jointly by a rehabilitation consultant and a respiratory consultant. Large MDT oriented mainly to rehabilitation. Strong research focus.
Site J (England)	Multi-tier service across a large urban area with significant socio-economic deprivation. Community clinic led by GP does in-person assessments; hospital-based clinic is led by a respiratory physician. Two MDTs meet on alternate weeks: tier 3 MDT discusses cases brought by the tier 2 team; tier 4 MDT (including multiple medical specialists) considers complex cases from across the region.

OT, occupational therapist; SLT, speech and language therapy; ME/CFS, myalgic encephalomyelitis/chronic fatigue syndrome.

All participating clinics offered multidisciplinary assessment and rehabilitation to patients referred from primary and secondary care. Patients with more challenging presentations were discussed at regular MDT meetings, usually online via Microsoft Teams. Some clinics were led by a GP or community-based allied health professional (‘tier 2’); some were hospital based and led by a consultant physician (‘tier 3’) and one included a regional (‘tier 4’) MDT meeting bringing multiple medical and allied specialists together to discuss complex cases.

To set up the quality improvement collaborative, we followed standard methodology for ‘breakthrough collaboratives’, adapted for online because of pandemic restrictions and geographical distance.^27^^,^^28^ Two-hourly meetings were held approximately 8-weekly (13 in total); they covered a range of topics based on an initial priority-setting exercise among participating clinicians. In addition, a separate patient advisory group undertook its own audit of current practice by sending a short survey to each clinic; it produced a priority list of topics based on this audit, the group's lived experience and wider knowledge. To ensure that we captured the priorities of actual clinic patients as well as those of clinic staff and patient advisory group volunteers, semi-structured interviews were also conducted with patient participants at each site (total 29), focusing on their clinic experience.

Prior to each online quality improvement collaborative meeting, the core research team undertook literature searches and circulated published evidence relevant to the topic being discussed. In many cases, sites were already working to improve the aspect of care prioritised. Clinical research fellows from each site (early-career doctors, nurses or allied health professionals), supported by the clinic lead (typically a consultant) collected and presented data from their own site (including anonymised patient cases and audits of structure, process and outcome), summarised the evidence base, and contributed to group discussions and goal-setting for improvements.

Meetings were video-recorded with consent and transcribed in real time by the Microsoft Teams software; key sections relevant to our analysis were later replayed on video and re-transcribed in full. All interviews were audio-recorded with consent and transcribed. Data were anonymised and stored in accordance with data governance protocols.

The LOCOMOTION quality improvement collaborative ran from November 2021 to May 2023; some sub-studies and audits continued to December 2023. Semi-structured interviews were held with clinical research fellows and other clinic staff (*n* = 30) between January and February 2023 to explore local challenges to improving quality.

All data were stored securely on encrypted University of Leeds and University of Oxford computers, in line with both institutions’ information governance protocols. Quantitative data were analysed using simple descriptive statistics in Stata Version 18; qualitative data were analysed thematically.^29^ For each priority topic, these data were combined using narrative synthesis (i.e. the story of the unfolding improvement effort over time was written, illustrated with any numerical data and any caveats and comments on missing data and variation among sites). Most qualitative findings from this large study, including results of in-person visits to clinics and online ethnography of MDT meetings between July 2022 and November 2023, are reported separately.^30^^,^^31^

Local quality improvement initiatives are generally written up in a structured way using the SQUIRE-2 guidelines.^32^ Because this was a UK-wide, multi-site initiative in which each local site had different priorities and constraints (e.g. staffing, resources, referral pathways), the SQUIRE-2 framework required some adaptation. We include the SQUIRE-2 checklist as a supplementary file (Table S1).

## Results

### Overview

The dataset for this paper consisted of the priority topic lists drawn up separately by clinicians and patients along with the merged list, evidence summaries and slide decks prepared for the quality improvement collaborative meetings, transcribed (anonymised) discussions from those meetings, collated audit data from clinics before and after any agreed changes were implemented, and transcripts of patient (*n* = 29) and staff (*n* = 30) interviews.

The merged topic list addressed by the quality improvement collaborative is shown in Table 2; clinicians’ and patients’ priorities mostly overlapped; key differences are mentioned in relevant sections below.

**Table 2:** Prioritised topics in the LOCOMOTION collaborative along with goals, objectives, approach and outcomes.

Topic	Agreed goal	Specific objectives and approach	Outcome
TOPIC 1: ACCESS	Everyone with long covid should be seen promptly. Those needing specialist assessment should be able to access it in a timely manner	1a. *Reduce waiting lists* by increasing clinic slots, streamlining pathways and addressing bottlenecks. 1b. *Prioritise the sickest patients* by developing and applying evidence-based referral criteria. 1c. *Address inequities of access* through targeted approaches for specific groups. 1d. *Inform and support GPs* so that they are confident to see and manage some long covid patients (see 2c).	Waiting time for first appointment was reduced from months to weeks in all sites, though this was partly due to reduction in incidence of new cases. Local clinics refined their referral criteria, pathways and prerequisite work-ups (e.g. required blood tests), but standardisation across sites proved difficult. Site-based initiatives to improve equity of access led to increased referrals for some but not all disadvantaged groups (see examples in text).
TOPIC 2: ASSESSMENT AND CARE PLANNING	Everyone with long covid should have a thorough, holistic initial assessment, including tests as needed to exclude serious complications	2a. *Define the core elements of a holistic clinical assessment* and ensure patients receive relevant elements as needed (usually, via multidisciplinary team care). 2b. *Define and implement protocols for ‘red flag’ symptoms* (e.g. indicating thrombotic complications), including key investigations and timely referral. 2c. *Inform and support GPs* by producing and disseminating guidance and an infographic on basic long covid assessment and management.	A multidisciplinary author team (including lived experience experts) synthesised evidence from research, current practice and patient experience, producing a guide and infographic.^33^ It included specific management advice for ‘red flag’ symptoms and advice on symptom control. The guide was widely accessed and disseminated among GPs and in patient online networks.
TOPIC 3: MONITORING	Patients’ progress should by systematically monitored using evidence-based measures	3a. *Select and standardise patient-reported outcome measures* (PROMs) for use in long covid clinics, taking account of what outcomes matter to patients. 3b. *Address burden of monitoring*, acknowledging that long covid patients may find repeated and lengthy questionnaires exhausting and demoralising.	A disease-specific PROM for long covid, C19-YRS, had already been produced and validated^21^^,^^34^; the collaborative and patient advisory group endorsed this measure for use across the LOCOMOTION sites. Further validation of C19-YRSm was undertaken.^35^ Uptake and use of C19-YRS and other validated PROMs (e.g. EQ-5D-5L) in participating clinics was limited by staff capacity and patients’ (fluctuating) capability and energy.
TOPIC 4: FATIGUE and TOPIC 5: COGNITIVE IMPAIRMENT	Management of fatigue and cognitive impairment (which often coexist) should be evidence-based, guided by symptoms and functional capacity, and attentive to fluctuations	5a. *Identify and summarise research evidence* on fatigue and cognitive impairment in long covid, including ‘crashes’, also known as post-exertional symptom exacerbation (PESE) and post-exertional malaise (PEM). 5b. *Align clinic protocols* with evidence and ensure all clinicians are aware and following them. 5c. *Inform and support GPs* by producing a guide and infographic on this topic.	Research from one LOCOMOTION site^36^ affirmed patients’ and therapists’ impressions that symptom-guided pacing activities (rather than ‘graded exercise’) can reduce episodes of PESE/PEM. Case discussions and joint meetings with patient lived-experience advisors underscored the importance of symptom-guided management and helped routinise this approach. A multidisciplinary team produced a guide and infographic on cognitive impairment.^37^
TOPIC 5: BREATHING DIFFICULTIES	All patients with continuing respiratory symptoms should be managed and monitored according to evidence-based guidance	5a. *Identify and summarise research evidence and guidelines* on respiratory complications of COVID-19. 5b. *Align clinic protocols* with evidence and ensure all clinicians are aware and following them. 5c. *Inform and support GPs* by producing a guide and infographic on this topic.	Discussion of case vignettes along with (sparse) research evidence improved understanding of how best to support patients with breathing difficulties. A synthesis and guide (with infographic) was produced, with special emphasis on the commonest respiratory manifestation of long covid, breathing pattern disorder.^38^
TOPIC 6: ORTHOSTATIC INTOLERANCE AND DYSAUTONOMIA	All patients with orthostatic intolerance and other manifestations of dysautonomia should be identified and managed in accordance with evidence	7a. *Identity and summarise research evidence* on orthostatic intolerance and dysautonomia in long covid. 7b. *Assess the prevalence of orthostatic intolerance* by prospectively testing all patients attending long covid clinics. 7c. *Align clinic protocols* with evidence and ensure all clinicians are aware and following them. 7d. *Inform and support GPs* by producing a guide and infographic on this topic.	A multidisciplinary author team synthesised evidence from research, current practice and patient experience, producing a guide and infographic.^39^ A prospective study of consecutive patients (n = 277) across 8 of the 10 LOCOMOTION clinics found the prevalence of PoTS to be 7% and orthostatic hypotension to be 8%.^40^
TOPIC 7: VOCATIONAL REHABILITATION	All long covid patients should receive evidence-based support to return to work if appropriate	8a. *Identify and summarise research evidence* on how to support long covid patients to return to work. 8b. *Align clinic protocols* with evidence and ensure all clinicians are aware and following them. 8c. *Inform and support GPs* by producing a guide and infographic on this topic	Discussion of cases revealed multiple challenges in vocational rehabilitation (see main text). A multidisciplinary author team, including two lived-experience experts (one an occupational health physician) synthesised evidence to produce a guide and infographic.^41^

PAG, patient advisory group. ITU, intensive care unit.

In some priority topics, it was possible to identify evidence-based guidance and benchmark current practice against this; in other topics, the focus of discussion in the meetings was on gaps or ambiguities in the evidence base and the implications for practice and research. Rarely, the quality benchmark was a definitive randomised controlled trial or other high-quality evidence; more commonly in this new condition, it was what has been called ‘potentially better practice’—meaning, custom and practice in leading clinics.^42^

### Access, referral criteria and inclusivity

Patients’ top priority for quality improvement was the waiting time for an appointment. When this study began (September 2021), most clinics were struggling to deal with a backlog of referrals (waiting times varied from 9 weeks to 1 year), which had several inter-related causes. These included the high volume of need following several waves of the COVID-19 pandemic (see Introduction); lack of ring-fenced funding for long covid services in Scotland and (at secondary care level) Wales; overly complex pathways; and limited understanding and low confidence among GPs about long covid, resulting in few patients being assessed or managed in primary care before they were referred.

The backlog of people waiting to be seen was steadily cleared through a combination of attention to local bottlenecks, triage, developing and disseminating referral criteria, streamlining pathways (e.g. shifting from ‘GP referral→in-person clinical assessment→referral to online rehabilitation programmes’ to ‘GP referral→direct access to online rehabilitation programmes while waiting for clinical assessment’), and supporting local GPs to become confident to manage some patients themselves. Waiting times were reduced from months to weeks in all participating clinics over the study period, though not all improvement can be attributed to the collaborative since incidence of new long cases fell over this period^5^; nationally, the proportion of long covid referrals seen within 6 weeks of referral increased from 31% in September 2021 to 58% in November 2023; the proportion waiting more than 14 weeks fell from 33% to 16% over the same period).^43^

Another access issue identified was inequity. Some sites had data suggesting that people from minority ethnic groups and socio-economically deprived areas were less likely to be referred, despite some evidence of disparities in long covid outcomes by deprivation, race and ethnicity.^44^^,^^45^ There were concerns that some disadvantaged groups were not aware of the service or would feel reluctant to attend, and that the predominantly online format of information discriminated against the less digitally-enabled. Various site-based initiatives were introduced. Site A, for example, worked with third-sector providers and specialist GP centres for homeless and refugee populations to raise awareness of long covid and available services. Site D worked with local low-income communities to create paper leaflets, posters and booklets to be distributed. Site E used a data dashboard to identify GP practices with low referral rates and then an outreach approach to those practices. Site F worked with third-sector providers and specialist GP centres for homeless and refugee populations as well as providers working with deprived, ethnic minority and learning-disabled patients, to raise awareness of long covid and available services. This site also piloted a third-sector referral pathway to enable referrals that were not entirely dependent on GP diagnosis. These initiatives were associated with improvements in referrals from some disadvantaged groups in some sites, but efforts to increase referrals from homeless and traveller communities did not produce measurable improvements.

### Assessment and care planning

When our study began, there was no consensus on the preferred work-up or standard care plan for patients presenting with suspected long covid. A major task for the quality improvement collaborative was to review the (sparse) research literature, explore and document current practice across participating LOCOMOTION sites and capture the patient experience on this topic. The resulting evidence synthesis underscored the importance of comprehensive assessment, beginning with a detailed history (documenting premorbid status and the timing, nature and severity of COVID-19 infection(s) and noting any rare but critical red flag symptoms) plus physical examination and blood tests as indicated.^33^ The cornerstone of long covid management is whole-patient rehabilitation along with symptom control, ideally with a single clinician providing oversight and relationship-based care.

In most participating sites, a prerequisite for accepting a GP referral for suspected long covid was that blood tests had been done to exclude alternative diagnoses. These typically included full blood count, electrolytes, liver and renal function, calcium and vitamin D, C-reactive protein, ferritin, B12 and folate, HbA1c, thyroid function, and (in some clinics) IgA. Many (though not all) patients therefore arrived with these tests completed. An initial attempt to standardise this pre-assessment panel was abandoned when it became clear that variation was often warranted and reflected local service needs and constraints (e.g. case mix, availability of particular tests). Similarly, aspirations to standardise the criteria for cross-referral to other specialties were dropped when it became clear that such criteria must be locally determined depending on which if any in-house specialist clinicians (e.g. cardiology, psychiatry, speech and language therapy) were available.

### Patient-reported outcome measures for monitoring and research

**Fig. 1:**
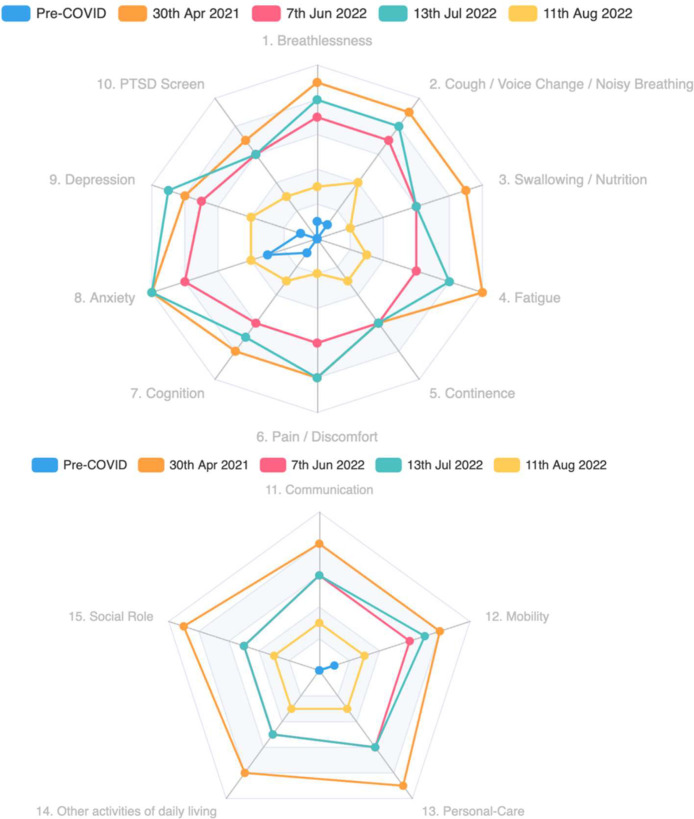
(a and b). Radar chart of patient responses to the Modified C19-YRS over time.

In the absence of a valid and reliable biomarker, the severity of long covid and the response to treatment is assessed using symptom checklists (patient-reported outcome measures or PROMs). The C19-YRS is a disease-specific PROM for long covid, initially developed and validated in 2020 and refined (by adding additional items) in 2022.^34^ The modified C19-YRS (see Fig. 1) asks about presence and severity of 10 symptoms—breathlessness, cough, fatigue, post-exertional malaise, cognition, pain, mood (including anxiety, depression and post-traumatic stress), altered smell/taste, palpitations/dizziness, and ‘other symptoms’ (including swallowing, allergy, tinnitus, menstrual upset and continence problems) and five areas of functional capacity (communication, mobility, personal care, other activities of daily living, social role).

Use of C19-YRS across participating clinics allowed comparison of clinic populations, but in order to compare with other long-term conditions a generic PROM (EQ-5D-5L^46^) was also used. In addition, more detailed symptom-specific PROMs (e.g. to characterise breathlessness, sleep problems, fatigue or altered smell in more detail) were sometimes judged appropriate for the patient's clinical care. While the information gleaned was often clinically useful, this had to be balanced against the burden of measurement on staff and patients and interpreted in the light of long covid's fluctuating course.

### Clinical controversies and dilemmas

Priority topics four through six addressed management of particular symptoms—fatigue, cognitive impairment, breathing difficulties and orthostatic tachycardia (which is sometimes but not always a symptom of dysautonomia). In each of these, the quality improvement collaborative meetings surfaced aspects of management for which the evidence from research was weak or contested, but on which much useful clinical discussion was held.

Key controversies in relation to fatigue and cognitive impairment (which tended to co-occur and co-vary) included how far to investigate to exclude differential diagnoses, how to handle requests for unproven therapies (e.g. hyperbaric oxygen, vagus nerve stimulation), and how best to manage fluctuations and avoid the well-described ‘crashes’ that can occur after physical, mental or emotional exertion.^33^^,^^37^ Even at the outset of our study, many therapists were already rejecting the ‘graded exercise’ approach (in which patients were encouraged to steadily increase their exercise levels regardless of symptoms) and advocating the ‘3Ps’: prioritising, planning and pacing^47^; by the end of the study period this symptom-guided approach was routine in all clinics.

Controversies around breathing difficulties also centred on how far to investigate patients, and with what tests. One key question, for example, was whether breathing pattern disorder was necessarily a diagnosis by exclusion (requiring extensive specialist tests) or whether it could be a positive diagnosis made on a careful history, clinical examination and basic lung function tests. Following case discussions and evidence review, we concluded the latter.^38^ Sharing case histories of patients with breathing pattern disorder across the collaborative surfaced an early finding that online breathing exercise programmes run by professional singers appeared to be highly beneficial and widely praised by patients (a finding that was subsequently confirmed in a randomised controlled trial^48^).

The investigation and management of palpitations and dizziness in long covid patients was another controversial clinical topic (see row six of Table 2). Some clinicians agreed with the patient advisory group that postural orthostatic tachycardia syndrome (POTS, defined as postural increase in heart rate without orthostatic hypotension and indicating possible dysautonomia) was likely being missed, while others felt that dysautonomia was rare in their clinics and overdiagnosed. A prospective prevalence study using the NASA Lean Test confirmed the important finding that POTS is not uncommon in long covid and sometimes presents without typical symptoms of orthostatic intolerance.^40^ Additional controversies included when and for whom to prescribe ‘off-label’ medication for POTS, how to manage patients in whom tachycardia or other potentially dysautonomic symptoms were precipitated by activities other than standing (e.g. eating, stress), and those in whom it was hard to tell which was the underlying issue—tachycardia or anxiety. Research evidence provided few clues on these questions and clinical views were polarised. Nevertheless, discussion across participating clinics helped map the state of knowledge, ambiguity and uncertainty in this contested topic.^39^

### Supporting patients to return to work

All clinics offered vocational advice and support and some offered formal vocational rehabilitation^49^ as part of the holistic management package; the quality improvement collaborative allowed all sites to learn from clinics with specialist expertise and research interests in this topic. While there was little evidence specific to long covid, wider evidence on vocational rehabilitation after prolonged infectious illness provided important insights—for example, that it is usually unwise to try returning to work until one is comfortable undertaking activities around the house (though return to work is possible before the patient is fully recovered) .^41^ Return-to-work support was particularly challenging in people with long covid who were self-employed or in insecure employment and for those who remained unable to do their previous full-time job after more than 6 months, especially when employers had fixed return-to-work policies, or where work patterns are incompatible with recommended adjustments (e.g. fixed-length shifts). Return in fatigued patients was often complicated by long exhausting commutes, trade-offs with home life (an apparently successful return occurring at the expense of family commitments), loss of income if return involved reduced hours, challenges of returning to a safety-critical occupation when not completely better (e.g. residual cognitive impairment or weakness), and recurrent relapses triggered by the physical and cognitive demands of work. Clinics specialising in vocational rehabilitation were sometimes able to engage directly with patients’ employers to advocate for specific adjustments to work roles.

## Discussion

This 2-year study of 10 long covid clinics participating in a quality improvement collaborative has produced four main findings. First, the agreed goal of accessible, holistic, multidisciplinary assessment and management has been met by widely differing service models and staff mix. Ring-fenced funding for long covid services in England produced some well-resourced clinics with a wide range of staff, and some teams in Scotland and Wales managed to develop similar (but less well-resourced) services with little or no ring-fenced funding. Second, even in the absence of high-quality evidence from randomised controlled trials, meeting regularly for case discussions and deliberation provides important learning for individuals and teams. Third, these collective deliberations, along with lived-experience input from patients, can generate important new research questions, some of which may be answered by prospectively collecting data within the clinics. Finally, while some patients with long covid are complex and require specialist investigation and management, many cases are straightforward and can be safely managed in community settings with support and guidance from specialist clinics (subject to resources and with attention to equity).

To our knowledge, this is the first national quality improvement collaborative reported for the new condition of long covid. Strengths include the diverse sampling frame (with sites from three UK jurisdictions and serving widely differing geographies and demographics, spanning primary and secondary care); presence of clinical research fellows in each clinic; and involvement of patient co-researchers and coauthors during the research and writing up.

Limitations of the study include an exclusive UK focus (transferability to other healthcare systems is therefore unknown); the self-selecting nature of sites (who may have represented the higher end of a quality spectrum); lack of definite evidence base for managing the condition; and competing demands on people's time and energy (the study was undertaken at a time when the UK National Health Service as a whole was under extreme austerity pressures).

## Conclusions

Four years after the first cases were described, long covid remains a common and disabling condition which is not widely understood and for which there is currently no cure. We have shown that important learning about a new and complex condition can be generated from ‘business as usual’ NHS clinics with input from lived-experience experts. The findings of this study underscore the latest commissioning guidance from NHS England (December 2023), which recommends multidisciplinary team care, tests and further referrals as indicated, needs- and symptom-based rehabilitation, supported self-management (with online and other resources directed at patients), integrated peer support and social prescribing, workforce training, and involvement of people with lived experience in design and evaluation of services.^25^ These principles and approaches may well apply to other post-infectious syndromes and should be noted in anticipation of the next pandemic.

**Summary box**

**What is known?**

Long covid (the protracted form of COVID-19 which follows acute infection in some patients) can be prolonged and debilitating. Specialist clinics were set up in 2021 to deal with this new condition.

**What is the question?**

Can an online quality improvement collaborative help optimise access, assessment, monitoring and management in long covid? What challenges do clinics face and how can they be addressed?

**What was found?**

1. The goal of accessible, holistic, multidisciplinary assessment and management was met by different services using different models and staff mix. Some services improved access for some disadvantaged populations
2. Most topics prioritised for quality improvement had a weak or contested evidence base, but even in the absence of high-quality research evidence, much learning and improvement occurred.
3. Collective deliberation about clinical controversies, along with discussions with patient advisory group representatives, generated new research questions, some of which were addressed through multi-site data collection.
4. While some patients require specialist input, straightforward cases can be assessed and managed by GPs with guidance and support from specialist services (subject to resources).

**What is the implication for practice?**

The findings support continuation of specialist long covid clinics along with linked rehabilitation services. The role of primary care in the ongoing management of long covid, and ways to improve equity of access, should be explored further.

Alt-text: Unlabelled box

## Funding

This work is independent research funded by the National Institute for Health and Care Research (NIHR) (long COVID grant, Ref: COV-LT2-0016). The views expressed in this publication are those of the authors and not necessarily those of NIHR of The Department of Health and Social Care. For more information about the LOCOMOTION study, please visit https://www.isrctn.com/ISRCTN15022307.

## Informed consent statement

Written informed consent was obtained from all subjects involved in the study. No patient-identifiable information is included in the manuscript.

## Ethics statement

LOng COvid Multidisciplinary consortium Optimising Treatments and servIces acrOss the NHS study is sponsored by the University of Leeds and approved by Yorkshire & The Humber–Bradford Leeds Research Ethics Committee (ref: 21/YH/0276) and subsequent amendments.

## CRediT authorship contribution statement

**Julie Darbyshire:** Writing – review & editing, Project administration, Investigation, Data curation. **Trisha Greenhalgh:** Writing – review & editing, Writing – original draft, Methodology, Investigation, Funding acquisition, Formal analysis, Data curation, Conceptualization. **Nawar D. Bakerly:** Writing – review & editing. **Kumaran Balasundaram:** Writing – review & editing, Data curation. **Sareeta Baley:** Writing – review & editing, Data curation. **Megan Ball:** Writing – review & editing, Data curation. **Emily Bullock:** Writing – review & editing, Data curation, Conceptualization. **Rowena Cooper:** Writing – review & editing, Data curation. **Helen Davies:** Writing – review & editing, Investigation, Data curation. **Johannes H. De Kock:** Writing – review & editing, Investigation, Data curation. **Carlos Echevarria:** Writing – review & editing, Data curation. **Sarah Elkin:** Writing – review & editing, Data curation. **Rachael Evans:** Writing – review & editing, Investigation, Data curation. **Zacc Falope:** Writing – review & editing. **Cliodhna Flynn:** Writing – review & editing, Data curation. **Emily Fraser:** Writing – review & editing, Investigation, Data curation. **Stephen Halpin:** Writing – review & editing, Investigation, Data curation. **Samantha Jones:** Writing – review & editing, Data curation. **Rachel Lardner:** Writing – review & editing, Data curation. **Cassie Lee:** Writing – review & editing, Methodology, Investigation, Formal analysis, Data curation. **Ashliegh Lovett:** Writing – review & editing, Data curation. **Victoria Masey:** Writing – review & editing, Data curation. **Harsha Master:** Writing – review & editing, Investigation, Data curation. **Ghazala Mir:** Writing – review & editing, Investigation, Data curation. **Adam Mosley:** Writing – review & editing, Data curation. **Jordan Mullard:** Writing – review & editing, Data curation. **Rory J. O'Connor:** Writing – review & editing, Data curation. **Amy Parkin:** Writing – review & editing, Investigation, Data curation. **Anton Pick:** Writing – review & editing, Data curation. **Janet Scott:** Writing – review & editing, Investigation, Data curation. **Nikki Smith:** Writing – review & editing, Data curation. **Emma Tucker:** Writing – review & editing, Data curation. **Paul Williams:** Writing – review & editing, Data curation. **Darren Winch:** Writing – review & editing, Data curation. **Conor Wood:** Writing – review & editing, Data curation. **Manoj Sivan:** Writing – review & editing, Methodology, Investigation, Funding acquisition, Data curation, Conceptualization.

## Declaration of competing interest

The authors declare the following financial interests/personal relationships which may be considered as potential competing interests:

Trisha Greenhalgh reports financial support was provided by National Institute for Health and Care Research. If there are other authors, they declare that they have no known competing financial interests or personal relationships that could have appeared to influence the work reported in this paper.

## Appendix Supplementary materials

Image, application 1

Image, application 2
