# Supplementary material for: Improving quality in adult long covid services: Findings from the LOCOMOTION quality improvement collaborative
Source: Clin Med (Lond). 2024 Aug 23;24(5):100237. doi: 10.1016/j.clinme.2024.100237 (PMC11421994; doi:10.1016/j.clinme.2024.100237)
Supplement: Supplementary file 1 [file mmc1.docx]

# SUPPLEMENTARY MATERIAL 2: SQUIRE-2 CHECKLIST

**Table S1.** SQUIRE-2 checklist for the LOCOMOTION Quality Improvement Collaborative (adapted from^1^)

| **Item** | **Requirement** | **How addressed** |
| --- | --- | --- |
| Title | Should indicate that this was an initiative to improve quality and state the clinical topic | Title includes the terms ‘quality improvement collaborative’ and ‘adult long covid’ |
| Abstract | Should be structured and include all key information, with searching and indexing in mind | Abstract is structured in IMRAD format. Key clinical topics covered in the QIC are listed |
| Introduction | This should justify why the quality improvement initiative was started | Introductory section follows SQUIRE-2 format, covering problem description, available knowledge (literature review), rationale (justification of the approach) and specific aims |
| Methods 1 (site descriptions) | The participating site(s) should be described | Table 1 lists the 10 participating sites and gives some background on their characteristics and history, with cross-referencing to supplementary material where these are described in more detail |
| Methods 2 (interventions) | Each intervention should be described in sufficient detail to replicate it. | The over-arching methodology (‘breakthrough collaborative’), including how specific improvement topics were prioritized, is described. Then, the 7 prioritized topics are listed (column 1 of Table 2). For each, we provide a brief description of the goal (column 2) along with the specific objectives and approach (column 3). |
| Methods 3 (outcome measures and contextual factors) | State how the impact of each intervention was measured *and* how the impact of contextual influences was assessed | Column 4 of Table 2 lists what outcomes were measured. A detailed ethnographic study of the contextual influences for each site is reported in separate qualitative papers^2 3^ and cross-referred to in the Results section |
| Methods 4 (analysis) | State how data were analysed, including drawing together qualitative and quantitative data and explaining variation between sites | We list the statistical package used for quantitative data and briefly describe how qualitative data were analysed thematically before preparing narrative summaries of each priority topic |
| Methods 5 (Under each sub-heading) | Provide details of ethics review and approvals | This is given in the first paragraph of the Methods section |
| Results 1 (description of the process) | Describe what happened at each step of the intervention | Details of the how the intervention for each priority topic was implemented is given in the text of the Results section, taking each topic in turn under subheadings |
| Results 2 (description of, and commentary on, outcomes) | Describe the outcomes, including comment on how successful the effort was (and suggest why) | Achieved outcomes are briefly listed in column 4 of Table 2; they are expanded on under each topic sub-heading in the Results section |
| Results 3 (explanation of variation among sites) | Compare how quality improvement efforts met with more or less success in different sites | Under each sub-heading, we briefly mention key elements of variation among sites. These site-to-site differences are the main focus of two in-depth qualitative papers^2 3^ and one quantitative paper^4^ published separately |
| Results 4 (additional comments) | Comment on (e.g.) unintended consequences or missing data | Missing data is discussed where relevant under each priority topic sub-heading (e.g. the need to balance researchers’ desire for data against the burden of measurement on staff and patients); see also additional papers for further exploration of consequences^2-4^ |
| Discussion 1 (summary) | Key findings should be summarised | Covered in paragraph 1 of Discussion |
| Discussion 2 (interpretation) | Findings should be contextualized in relation to existing literature | Covered in paragraph 2 of the Discussion |
| Discussion 3 (limitations) | List the key limitations, especially those which limit the generalisabilty of findings | Covered in paragraph 3 of the Discussion |
| Discussion 4 (conclusions) | Draw conclusions, including about usefulness, implications and next steps | Covered in a separate Conclusion section as per journal house style |
|  |  |  |

**References**

1. Ogrinc G, Davies L, Goodman D, et al. SQUIRE 2.0—Standards for Quality Improvement Reporting Excellence—revised publication guidelines from a detailed consensus process. *Journal of the American College of Surgeons* 2016;222(3):317-23.

2. Greenhalgh T, Darbyshire JL, Lee C, et al. What is quality in long covid care? Lessons from a national quality improvement collaborative and multi-site ethnography. *BMC Medicine* 2024(1):159.

3. Greenhalgh T, Darbyshire J, Ladds E, et al. Working knowledge, uncertainty and ontological politics: An ethnography of UK long covid clinics. *Sociology of Health & Illness*

4. Lee C, Greenwood DC, Master H, et al. Prevalence of orthostatic intolerance in long covid clinic patients and healthy volunteers: A multicenter study. *Journal of Medical Virology* 2024;96(3):e29486.
