# Supplementary material for: Improving quality in adult long covid services: Findings from the LOCOMOTION quality improvement collaborative
Source: Clin Med (Lond). 2024 Aug 23;24(5):100237. doi: 10.1016/j.clinme.2024.100237 (PMC11421994; doi:10.1016/j.clinme.2024.100237)
Supplement: Supplementary file 2 [file mmc2.docx]

# SUPPLEMENTARY MATERIAL: NARRATIVE SUMMARIES OF THE TEN LOCOMOTION CLINICS

Most of the 10 participating clinics in LOCOMOTION had been established in 2020 (two began in 2021) to meet new demand from patients who were not recovered following acute covid-19. Despite some commonalities in origins, the clinics had different historical path-dependencies, geographical and material settings, staffing structures, patient pathways and case mix.

Broadly speaking, there were three underlying models for long covid clinics. These models were not mutually exclusive, and different clinics in our sample had characteristics of all three.

The **‘respiratory follow-up’** model (tier 3) emerged to monitor patients who had been hospitalized (and in some cases ventilated) for severe acute covid-19. These patients typically had persisting respiratory symptoms (cough, shortness of breath, abnormal breathing pattern), visible lung damage on chest X-ray or CT scan, and reduced lung function. Such clinics initially followed early guidance published nby the British Thoracic Society (which recommended following patients up with a chest X-ray at 12 weeks).^1^ They focused primarily on monitoring the patient’s recovery with lung function tests and serial imaging, as well as managing any coexisting respiratory disease (e.g. asthma, COPD).

The **‘rehabilitation’** model (tier 3) emerged to deal with patients whose symptoms were more generalised; they were often dominated by fatigue and functional impairment. Patients were usually referred by their GPs after their recovery proved more protracted than anticipated. The first appointment at such a clinic was usually lengthy and thorough: it began with the clinician documenting the patient’s medical history (including but not limited to when and how severely they had had covid-19) and their current symptoms and functional level in great detail, often using PROMs. This assessment, along with a physical examination and (if indicated) blood tests and imaging, was partly oriented to excluding serious complications or coexisting conditions. Once this had been done, the patient was given advice oriented to optimising convalescence and reducing setbacks, such as the ‘three Ps’ (pacing, prioritising and planning), along with psychological and practical support. Vocational rehabilitation was offered in some of these clinics to help the patient plan and negotiate their return to work (e.g. phased return, modified role). Patients were invited to join various group programmes such as ‘understanding long covid’, ‘fatigue management’, ‘restoring normal breathing pattern’ (including a popular online programme offered by opera companies), ‘mindfulness’ and ‘dealing with the emotional aspects of long covid’. All these programmes were mostly held online via group video clinic. The rehabilitation model anticipates that, with support, many if not all patients will eventually return to their previous levels of activity and performance. Serial PROMs help chart the patient’s progress from baseline.

There is also a **‘GP/community care’** model (tier 2), led by a GP with special interest or allied health professional (typically an occupational therapist). Such clinics take referrals from fellow GPs and have strong links to community rehabilitation services. Their focus is on holistic assessment, providing affirmation and confirming the diagnosis; signposting more straightforward cases to peer support and self-management resources; and investigating and referring where indicated to exclude serious complications (including those due to cardiac, pulmonary and neurological organ damage).

As mass vaccination reduced the severity of acute covid-19 for most people, the patient population in all long covid clinics has progressively shifted to include fewer ‘post-ICU [intensive care unit]’ patients (in whom respiratory symptoms almost always dominated), and more people referred by their general practitioners who had not been hospitalized (in whom fatigue, brain fog and palpitations were often the most troubling symptoms). Services that had begun as respiratory follow-up shifted to embrace a wider rehabilitation function. Following the cessation of available testing for covid-19, many patients were referred without a positive test (and sometimes without a clear history of a covid-like illness). Hence, over time, some long covid clinics have become less easily distinguishable from chronic fatigue services.

A fourth model, the **‘specialist care’** (tier 4) model, comprising a tertiary referral service for patients suspected of having complications, particularly end-organ damage, or complex comorbidities. For example, some cardiology services take referrals for suspected postural orthostatic tachycardia syndrome (POTS); some neurology services accept patients with severe neurocognitive symptoms; some allergy services accept cross-referrals for patients suspected of MCAS (mast cell activation syndrome); and so on. At the time of our study, these specialist services were not evenly distributed across the country and were unavailable in many settings, though many patients knew of their existence from online peer support groups.

At the time of our fieldwork (2021-2023), the 10 clinics were running various combinations or variants of respiratory follow-up, rehabilitation, community care and specialist investigation. We outline these briefly below as we describe the ten LOCOMOTION sites.

**Site A (England)** is based in a large conurbation in central England; it serves a large and dispersed population and is linked to a teaching hospital. It includes a GP-led tier 2 clinic, based in the community healthcare trust, with a core MDT including a rehabilitation consultant, nurses, physiotherapists, occupational therapists (OTs), a dietitian and a psychologist. The team operates with a hybrid model (typically by telephone or video assessments to assist with energy preservation, limit travel time, improved accessibility with less time off work as well as relatives being able to join) but will also bring patients in for a face-to-face assessment if indicated (e.g. for physiological testing, breathing pattern retraining or if other digital challenges arise). The community team can also visit patients in their homes if required and appropriate for 1:1 specialist fatigue management. Initial assessments take up to 90 minutes and follow-up assessments typically take 30 minutes. In addition to these detailed holistic assessments, the clinic is able to refer to specialist services including vocational rehabilitation, speech and language therapy, musculoskeletal physiotherapy, talking therapies, pulmonary rehabilitation, continence team as well as to group programmes covering fatigue management and better breathing. The clinic also has established links with the chronic fatigue service to provide a direct advisory service as well as a fast-tracked route into that service. There is also a tier 3 clinic based in the teaching hospital, which began as a post-ITU respiratory follow-up service and is led by a respiratory consultant; this service refers to the same rehabilitation teams and may cross-refer patients to the tier 2 clinic as appropriate.

**Site B (Wales)** is a small hospital-based tier 3 clinic run by a consultant chest physician with support from a respiratory specialist doctor in training and a physiotherapist. Because it is not based in England it does not attract ring-fenced funding (though some ring-fenced funding was available for a community-based long covid service). The clinic accepts referrals from primary and secondary care. All referrals are triaged by the consultant. There is no formal MDT support but input is available on request from the community-based rehabilitation team.  Most initial consultations are face-to-face; follow-up appointments are virtual or face-to-face as appropriate.

**Site C (England)**is a tier 2 clinic led jointly by a GP (with an interest in infectious disease) and an OT. It was established initially as a generalist ‘assessment-only’ service but evolved early on to include treatment as well. It was developed and is assisted by a wide range of other professionals including a specialised rehabilitation coordinator, speech and language therapy (SALT), dietetics, pulmonary rehab, clinical psychology, mental health and community-based physiotherapy and OT. It has close links with a chronic fatigue service and a pain clinic that have been running in the locality for over 20 years. The clinic is physically located in a low-rise building on the industrial outskirts of a large town, sharing office space with various community-based health and social care services. Clinical staff work entirely virtually and consult either from home or from a small side office in the community trust building, by telephone or video. Following an initial one-hour triage telephone consultation by one of the team, patients are either discharged back to their general practitioner with a detailed management plan or referred on to one of the in-house community services noted above. Patients with ongoing or unexplained medical issues are referred to and seen by the GP, who also does a holistic detailed review and can organise further investigations or refer patients onwards to respiratory, cardiology, ENT or neurology for more specialised review if required.  Complex patients are discussed at the weekly MDT. There is no tier 3 long covid clinic in this site.  This arrangement evolved to address a particular problem in this locality—that many patients with long covid were being referred by their general practitioner to multiple specialties (e.g. respiratory, neurology, fatigue), leading to a fragmented patient experience, unnecessary specialist assessments and wasteful duplication. The generalist assessment by telephone is oriented to documenting what is often a complex illness narrative (including pre-existing physical and mental comorbidities) and working with the patient to prioritize which symptoms or problems to pursue in which order.

**Site D (Scotland)** is a dispersed team who deliver a mainly virtual service and meet in-person once a month. The lead is a clinical psychologist, and the MDT also includes an infectious disease physician, GP, physiotherapist and vocational OT, along with IT support.  The infectious diseases specialist consultant also sees patients with Lyme Disease and takes a special interest in post-treatment Lyme disease.  ​Like site B, this service was not eligible for England’s ring-fenced funding when it was established, so is smaller and less well-resourced. Some ring-fenced Scottish Government funding has however recently become available.  The clinic serves a wide geographical area, much of it rural and remote. The clinic is run on a rehabilitation model with an initial virtual consultation (by video or phone) and a panel of investigations to assist in identifying alternative or concurrent diagnoses.  Blood tests and other investigations are facilitated by a network of phlebotomy and test centres across the region, and in remoter areas by local GPs.  All patients also undergo a NASA lean test at home. There is a small capacity for face-to-face clinic reviews when required. At the weekly (virtual) MDT meetings, a comprehensive list of patients is reviewed. This list includes new patients referred into the service for whom decisions about first contact are made. These contacts can be with any of the team and the focus of the appointment will depend on the details in the referral. The team will also discuss patients in the service who either require more in-depth support or who they think should be discharged. Not all patients referred have long covid. Although symptoms may have worsened since a covid-19 infection, several have symptoms that pre-date such infection.

**Site E (England)** is a tier 3 clinic located in the chest and allergy outpatient department of a major teaching hospital. It was set up by a respiratory physician with an interest in long covid, but because of other demands on that person’s time, its leadership is now shared across multiple respiratory consultants in rotation. The clinic’s initial ethos and rationale had been ‘respiratory follow-up’, with strong emphasis on monitoring lung damage in patients who had been hospitalised via repeated imaging and lung function tests and in ensuring that patients received specialist physiotherapy to ‘re-learn’ efficient breathing techniques. Over time, the clinic has shifted to being an *assessment* service in which most new patients are GP referrals who were never admitted to hospital with acute covid-19. The clinic has tried to accommodate the shift towards a more fatigue-predominant case mix by embracing the wider aspects of rehabilitation; it has good links with the community rehabilitation service and also well-established referral pathways to other services, including a tier 4 cardiology clinic specialising in POTS (postural orthostatic hypotension syndrome). At the time of our fieldwork, the clinic began with a brief MDT meeting in which staff discussed the patients they were about to see. Then, each patient was seen in turn by a physician, psychologist, occupational therapist and respiratory physiotherapist (half an hour each) before all four staff reconvened in a face-to-face MDT meeting to form a plan for each patient and ensure that letters back to the patient’s GP include all key information about tests and onward referral. Whilst a wide range of patients with diverse symptoms are now seen in this clinic, there remains a strong focus on respiratory pathology (e.g. tracking improvements in lung function and ensuring that coexisting asthma is optimally controlled).

**Site F (England)**, one of the first long covid services in the UK, was set up in the summer of 2020 after local evaluation demonstrated the high frequency of longer lasting symptoms. Rehabilitation medicine physicians as well as therapists were centrally involved in the design and therefore from the outset this service was more oriented to rehabilitation, including vocational rehabilitation to help patients return to work. Rather than an assessment clinic model, the service held a caseload and worked with individuals over time, through face to face, virtual and group contacts. Symptom-based investigations screen for medical problems but there was less emphasis on monitoring lung function or pursuing respiratory comorbidities. The service is community-based and operationally led by two clinical co-ordinators from a physiotherapy background, and includes a wide MDT of occupational therapists, a psychologist, dietician and therapy assistants, and consultants in rehabilitation medicine, respiratory medicine and cardiology. Staff offer each patient a full physical, cognitive and psychological assessment and provide support to identify meaningful functionally focused goals, while staying within safe energy limits. Research has been thoroughly integrated in the service providing many opportunities for patients to be involved in studies and generating new evidence.^2^

**Site G (England)** offers a tier 3 service based in the outpatient department in a busy acute university hospital located on the outskirts of a city. Like site F, it was one of the first post-covid clinics to be set up in the UK (early June 2020), originally to follow-up the high numbers of people with severe acute COVID-19 across the wider NHS Trust. The purpose was to both investigate the post-covid sequelae and provide healthcare via a holistic assessment to identify medical, rehabilitation and mental health needs, alongside social support needs. The service was adopted as an NHS England Long Covid service in Oct 2020, thereby expanding access to adults with long covid managed in the community for their initial illness. The service is led by a consultant respiratory physician and a respiratory Advanced Nurse Practitioner (ANP). All patients hospitalised with COVID-19 are reviewed by the long covid service. GPs refer to this service via a ‘single point of access’ (i.e. patients can only access long covid rehabilitation after specialist review). The referral form directs GPs to relevant resources that might be helpful for them or their patients such as the 'yourcovidrecovery' website. The form includes a brief description of the service eligibility criteria and suggests basic investigations to be performed prior to referral. Initial assessment is a one-hour telephone structured review including validated health questionnaires by a team of respiratory nurses. The diagnosis of long covid is usually confirmed (when it is excluded, patients are either referred back to GP or occasionally to an appropriate specialist). Using shared decision making, the subsequent pathway may involve further investigations before a (briefer) in-person appointment at the long covid clinic and/or patients may be referred to rehabilitation, mental health services or specialist respiratory physiotherapy for breathing pattern disorder. The long covid service also involves a weekly multi-disciplinary virtual team meeting for half an hour involving respiratory, cardiology, liaison psychiatry and neurology consultant physicians, respiratory specialist registrar, members of the rehabilitation team (nurses and physiotherapists), the respiratory long covid nurses and dedicated administrator (and, more recently, long covid GP champions). The long covid clinic is delivered by consultant respiratory physicians including a specialist consultant in interstitial lung disease, a consultant cardiologist and a respiratory ANP. The MDT generally seek and bring in further specialist advice where possible rather than referring patients to other specialities. This is to reduce burden both to the patient and the healthcare system. Patients are discharged when the diagnoses are secure and interventions underway with a trajectory of improvement. There is very limited access to specialist chronic fatigue services and memory clinics for young people in this setting.

**Site H (England)** was originally established as a tier 3 respiratory follow-up clinic based in a large teaching hospital to assess patients who had received ventilatory support during a hospital admission. It evolved to become a comprehensive assessment clinic with referral pathways to other secondary care services. Referrals were accepted from GP, Occupational Health and secondary care; all patients were initially seen face-to-face in multiple specialist appointments (each lasting 15-30 minutes) with respiratory physician, infectious disease physician, physiotherapist and psychology; all had bloods and full lung function testing, and were then assessed for whether further investigation and referral was needed. Those identified as needing further rehabilitation could be referred to the Post COVID rehab team which offered specialist physiotherapy, OT, dietician, psychology and speech and language input with approximately equal numbers of clinic appointments, home visits and group classes.  This clinic was later amalgamated into the local chronic fatigue and myalgic encephalomyelitis (CFS/ME) service, which had less capacity for holistic assessment but greater capacity to offer ongoing treatment and rehabilitation. The primary purpose of discussion in MDTs is to ensure that letters to patients and their GPs include all relevant details to initiate the referral process and ongoing support. A specialist investigation (tier 4) clinic located in the same city is run by a cardiologist with a special interest in dysautonomia and inflammatory coagulopathy. This clinic takes referrals from the regular long covid service as well as from general practitioners and other hospital consultants.

**Site I (South-East)** is a tier 3 service based in the Respiratory outpatient department on a large hospital site, co-located with various university research institutes. It is a collaborative service with two trusts working in parallel to deliver multidisciplinary and holistic care and incorporates a clinic-based assessment service with community-delivered rehabilitation. It is led jointly by a respiratory consultant and physiotherapist. The Long Covid team comprises consultants from respiratory and rehabilitation medicine with input from rheumatology and sports and exercise medicine. Allied professionals include respiratory specialist physiotherapists, occupational therapists with specialism in chronic fatigue management (including ME/CFS), a clinical psychologist and community psychological support services. Patients are referred by GPs using a specific proforma that includes information on symptom burden and functional impairment. They are triaged to the clinic to be seen initially by respiratory or rehab doctor, depending on symptoms, or directly to the community rehab team.  Consultations are scheduled for 40-minutes, are in-person and involve assessment and formulation of a management plan. Patients then typically go on to see allied professionals during the same appointment, who provide individualised support and invite the patient to join various rehabilitation programmes (mostly but not exclusively online). Each week there is a multidisciplinary meeting following the clinic to discuss optimal patient management and a fortnightly online MDT focused around patients with more complex rehabilitation requirements. The clinic has a strong rehabilitation focus and is keen to avoid ‘medicalising’ patients with unnecessary investigations, referrals or medicines. It also has a strong research ethos and is participating in various multi-site collaborative studies (in addition to LOCOMOTION); at their initial assessment visit, all patients are offered a menu of potential studies to join.

**Site J (England)** is part of large coalition of clinics on multiple sites, located in a city serving a large population with significant socio-economic deprivation. The multi-tier service includes an in-person tier 2 assessment clinic run by two GPs with special interest in long covid; it receives referrals directly from primary care and is linked to a community-based rehabilitation clinic led by specialist physiotherapist and supported by an OT, speech and language therapist, dietician, healthcare assistant, and administrative staff. This tier operates a rehabilitation model, with various group programmes around fatigue management, breathing and return to work. Cases are discussed in the weekly tier 2 MDT meetings, with patients referred from the assessment clinic into the rehabilitation clinic as appropriate. More complex cases are seen within the hospital-based tier 3 service, which is led by a respiratory physician, following a discussion at the tier 3 MDT (which also includes long covid services joining from elsewhere in the coalition). This could include assessment by a chest physician, a cardiologist, psychologist, or chronic fatigue specialist based on need. There is also a tier 4 MDT to which particularly complex cases can be further escalated; following discussion within this MDT, the case can then be seen by the appropriate specialist(s), for example neurologist, neuro-rehabilitation specialist, chronic fatigue specialist, or specialist liaison psychiatrist. Further input via tier 4 can be accessed as needed from other specialists including immunology, gastro-enterology or ENT. Both tiers 3 and 4 MDTs run virtually on alternate weeks.

1. British Thoracic Society. COVID-19: information for the respiratory community. London: BTS 2022 (last updated 18th January). Accessed 2nd June 2022 at <https://www.brit-thoracic.org.uk/covid-19/covid-19-information-for-the-respiratory-community/>.

2. Parkin A, Davison J, Tarrant R, et al. A multidisciplinary NHS COVID-19 service to manage post-COVID-19 syndrome in the community. *Journal of primary care & community health* 2021;12:21501327211010994.
